# Supplementary material for: A colloidal viewpoint on the sausage catastrophe and the finite sphere packing problem
Source: Nat Commun. 2023 Nov 30;14:7896. doi: 10.1038/s41467-023-43722-0 (PMC10689752; doi:10.1038/s41467-023-43722-0)
Supplement: Supplementary file 1 — Supplementary Information [file 41467_2023_43722_MOESM1_ESM.pdf]

A colloidal viewpoint on the sausage catastrophe and the finite sphere packing problem

Supplementary Information

Susana Marín-Aguilar,[1,*] Fabrizio Camerin,[1,2,†] Stijn van der Ham,[3]
Andréa Feasson,[3] Hanumantha Rao Vutukuri,[3,‡] and Marjolein Dijkstra[1,2,§]

[1]*Soft Condensed Matter & Biophysics, Debye Institute for Nanomaterials Science, Utrecht University, Princetonplein 5, 3584 CC Utrecht, The Netherlands*
[2]*International Institute for Sustainability with Knotted Chiral Meta Matter (WPI-SKCM²), Hiroshima University, 1-3-1 Kagamiyama, Higashi-Hiroshima, Hiroshima 739-8526, Japan*
[3]*Active Soft Matter and Bio-inspired Materials Lab, Faculty of Science and Technology, University of Twente, PO Box 217, 7500 AE Enschede, The Netherlands.*

Additional details on the state diagram

In Fig. S1, we present the state diagram as shown in the main text, where the symbols are colored according to the packing fraction η of the colloids in the vesicle for the various state points. This state diagram confirms that when the surface area of the vesicle is maintained constant for a specific N, the reduced volume ν is directly proportional to the packing fraction of the vesicle η. For the calculation of the packing fraction η, the volume of the vesicle is estimated by means of its surface mesh with the alpha-shape method of the OVITO software, which provides a three-dimensional surface representation of the vesicle [1, 2]. This method relies on a Delaunay tessellation constructed on the basis of the input particle coordinates and uses a probe sphere with prescribed radius $R_{probe} = 10 - 16\sigma$ (depending on the size of the vesicle) to assign each tetrahedral element to a region of space, of which the volume is calculated.

In Figure S2, we present representative simulation snapshots illustrating linear, planar, and cluster configurations. Additionally, Table S1 provides a summary of the main parameters, including the size of the membrane in its initial spherical state, its surface area, and the range of solvent particle densities employed to explore various conformations.

Transformation of the particle packings

To demonstrate the robustness of our experimental method, we show the transition of particles from a linear arrangement to a cluster state by precisely controlling the surface area-to-volume ratio of the vesicle. We induce transitions between the three distinct conformations by gradually increasing the vesicle volume while keeping the membrane surface area constant. We achieve this through a step-wise reduction in the osmolarity of the external solution, leading to a vesicle volume expansion. Particles in GUVs are initially arranged linearly, then they are subjected to an osmotic shock through the sequential addition of water outside the vesicle solution. In a typical experiment, we add a total of 50 μL of Milli-Q water in increments of 10 μL to the 40 μL vesicle solution. The first addition induces transition from the sausage to the plate conformation. The second addition transforms the plate to a cluster arrangement. Finally, the last three additions progressively transform the vesicle into a more spherical shape. We observe that each transition took place approximately 3-4 minutes following each addition.

A similar procedure is done in simulations, where we gradually change the shape of the vesicle by removing solvent particles, thus changing the density of the solvent. This allows us to observe the transformation from cluster to linear conformation. Fig. S3(a-d) show time-lapsed 2D overlaid fluorescence and bright-field microscopy snapshots of the cluster-to-linear transformation. Meanwhile, Fig. S3(e-h) depict the corresponding snapshots from simulations by changing the solvent density ρ_{sol}. The dependence of the vesicle volume V_v as a function of ρ_{sol} is displayed in Fig. S3(i).

Generation of clusters

To investigate the possibility of observing the sausage catastrophe in a flexible vesicle, and identifying cluster conformations of spheres that pack better than the linear arrangement, we equilibrate fluid vesicles of various sizes in such a way that a packing fraction of $\eta \approx 0.4$ is achieved for varying number of colloids N. This is the highest packing fraction we can reach by randomly adding spheres to the vesicle without breaking the meshless network. After adding the spheres, the system is allowed to equilibrate. The colloids and vesicle are subject to the interaction potentials described in the Methods section. Table S2 presents a summary of the vesicle sizes employed for a particular range of N.

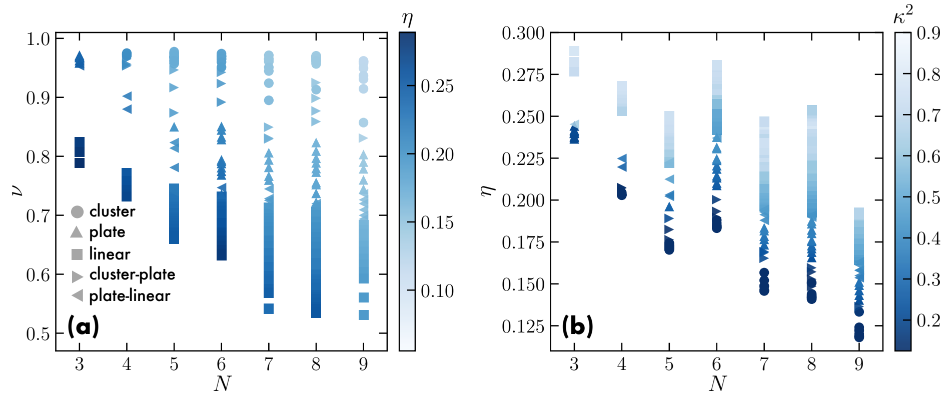

FIG. S1. (a) State diagram of colloidal hard spheres enclosed in a fluid vesicle as a function of the number of colloids N and the reduced volume $\nu = V_v/V_s$ with V_v the volume of the vesicle and V_s the volume of a sphere with the same surface area as the vesicle as obtained from simulations. For a specific N, the surface area of the vesicle is maintained approximately constant. Square symbols are for linear conformations, triangles for plates, and circles for cluster conformations, while right and left triangles represent bistable states. Symbols are colored according to the packing fraction η of the colloids inside the vesicle. (b) State diagram of colloidal hard spheres enclosed in a fluid vesicle as a function of the number of colloids N and packing fraction η. Symbols are the same as in (a).

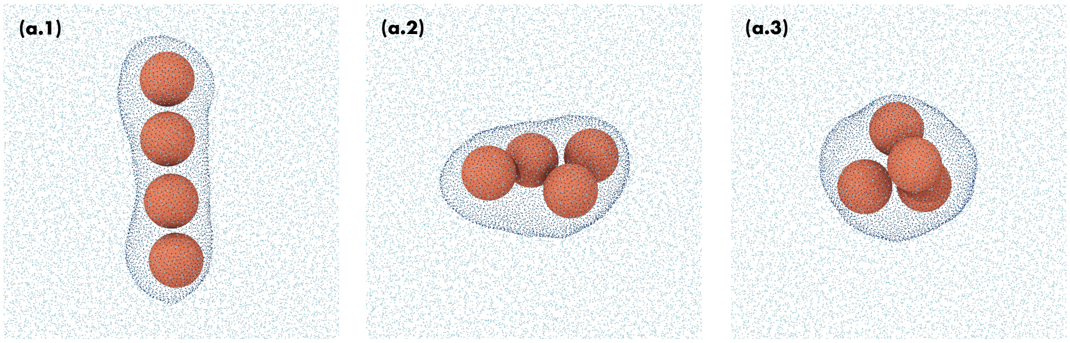

FIG. S2. Typical configurations of linear, planar and cluster conformations of colloids in a fluid vesicle obtained from simulations with the outer solvent exerting a pressure on the vesicle. The number density of solvent particles is $0.0360, 0.0341$ and $0.0327\sigma^{-3}$, respectively. Note that for visual clarity the vesicle beads and the solvent particles have a smaller size than the actual ones used in the simulations.

Optimization protocol to reach the hard-sphere limit of the clusters

The convex-hull packing fraction of the clusters reported in Figure 3 of the main text is obtained after a series of energy minimization steps in which the interaction potential between the colloids becomes more hard-sphere-like. To accomplish this, the configurations are subjected to a sequence of energy minimizations using the FIRE algorithm [3], regardless of whether they are obtained from the database [4] or generated from simulations involving colloids interacting with the repulsive WCA potential. In each step, a $2\alpha - \alpha$ LJ potential is used, with the exponent α increasing at each minimization step, causing the potential to become progressively steeper, with its minimum tending towards the sphere contact distance. Typically, α is varied from 6 to 140 in eight subsequent steps. Fig. S4 shows typical configurations obtained from simulations in which we visually describe the process of obtaining a cluster.

Convex hull

To obtain the convex-hull packing fraction η_{ch} of the generated clusters, we first tessellate the surface of the constituent spheres with a certain number of points N_{points}. We then determine the convex hull of these points [5] and the volume enclosed, and thus the packing fraction of the cluster within the convex-hull. This protocol is depicted in Fig. S5. In Fig. S6, we demonstrate how the packing fraction varies with N_{points}. As the calculated packing fraction only varies on the fourth decimal for $N_{points} > 10^5$, we use $N_{points} = 10^5$ for the remainder of the analysis.

Moreover, to further validate our approach, we com-

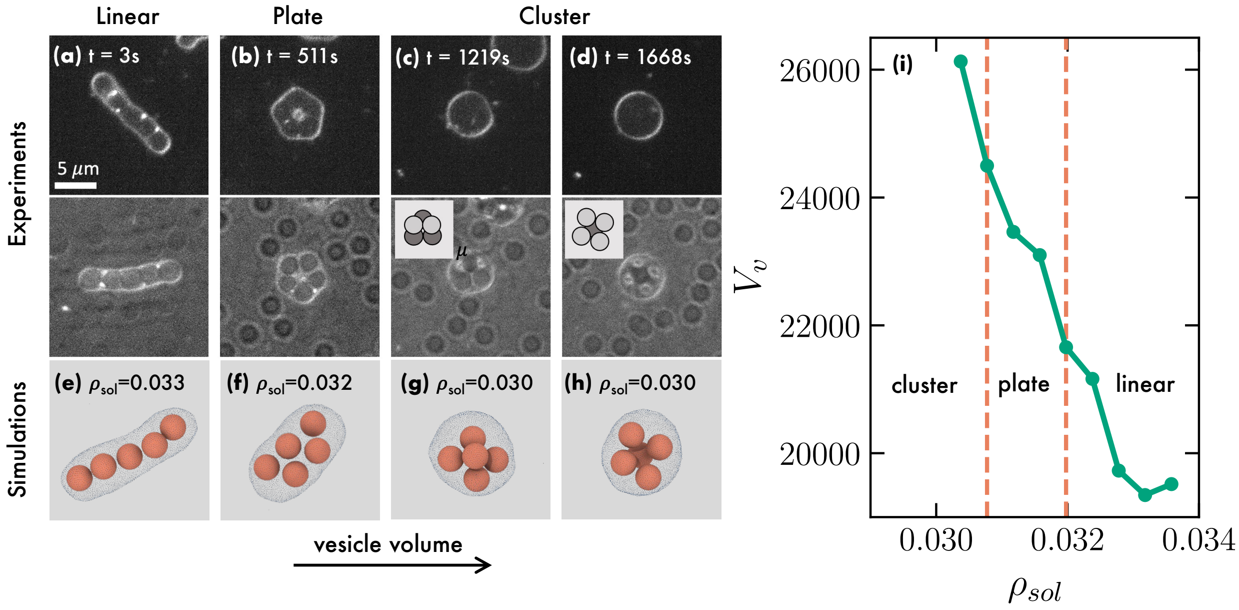

FIG. S3. (a-d) Sequence of time-lapse images from 2D fluorescence (top row) and overlaid fluorescence and bright-field (middle row) microscopy showing the transition from linear to cluster conformation as osmotic imbalances are induced in a vesicle containing five particles. (e-h) Corresponding simulation configurations generated by systematically changing the outer density solvent ρ_{sol}, as reported in the Figure in units of σ^{-3}. (i) Dependence of the vesicle volume V_v on ρ_{sol} in simulations. Dashed lines are guides-to-the-eye for distinguishing the transitions between the three different configurations.

N	$Z[\sigma]$	$A_v[\sigma^2]$	$\rho_{linear}[\sigma^{-3}]$	$\rho_{planar}[\sigma^{-3}]$	$\rho_{cluster}[\sigma^{-3}]$
3	30	2500.2±15.16	0.0364 - 0.0381	0.0331 - 0.0366	-
4	35	3364.82±9.29	0.0347 - 0.0363	-	0.0330 - 0.0322
5	40	4379.14±15.26	0.0328 - 0.0364	0.0312	0.0279 - 0.0309
6	42.5	4737.69±22.21	0.0328 - 0.0364	0.0310 - 0.0325	0.0293 - 0.0303
7	47.5	6166.72 ± 96.25	0.0307 - 0.0368	0.0286 - 0.0299	0.0261 - 0.0277
8	50	6823.54±24.53	0.0295 - 0.0368	0.0267 - 0.0288	0.0232 - 0.0258
9	55	8389.86±112.02	0.0280 - 0.0324	0.0240 - 0.0255	0.0197 - 0.0233

TABLE S1. Simulation parameters for the simulations: number of colloids N, diameter of the initial spherical vesicle Z in which the colloids are enclosed, average surface area of the vesicle A_v, and density $\rho_x = (N_x/L^3) \times (L^3/\sigma^3)$ with N_x the number of solvent particles outside the vesicle and L the side of the simulation box, for obtaining x = linear, planar and cluster configurations. Bistable states are obtained for densities in between the ones indicated in the Table and for $N = 4$ we do not identify fully stable planar configurations.

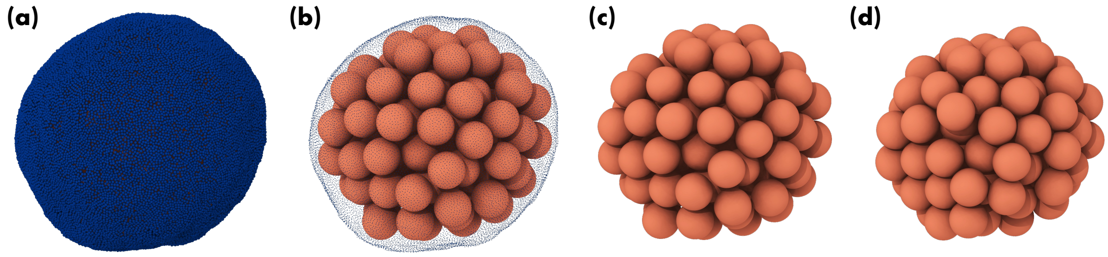

FIG. S4. Typical configurations obtained from simulations in which we show (a) the full vesicle, (b) the vesicle enclosing the cluster of colloids interacting via a WCA potential, (c) the cluster of colloids and (d) the cluster after having performed a series of energy minimization steps to make the interaction potential more hard-sphere-like.

pare η_{ch} of linear arrangements in the range $N = 1-100$

with the theoretical packing fraction of the linear con-

N	$Z[\sigma]$
10-11	40
12-16	45
17-21	50
22-30	55
31-46	60
47-63	65
64-79	70
80-97	75
98-118	80
119-142	85
143-150	90

TABLE S2. Range of the number of colloids N added to an initial spherical vesicle with diameter Z to study the formation of clusters in simulations.

formation $\eta_{lin} = 2N/(2+3(N-1))$ obtained from the volume of the spherocylinder with N particles, as $\Delta\eta = \eta_{lin} - \eta_{ch}$, as shown in Fig. S7. We find that the precision is again on the fourth decimal digit.

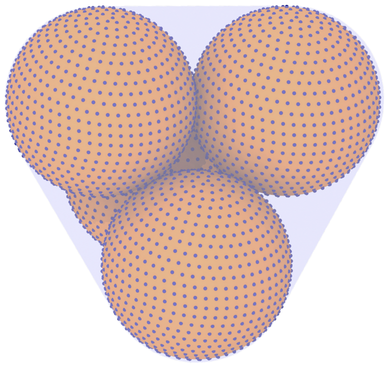

FIG. S5. Tessellation procedure for an example tetrahedral cluster where small spheres are added to the surface of the colloids. The convex hull and packing fraction of the cluster is calculated using these points.

Clusters from the database

In Fig. S8, we show from different perspectives some of the clusters as obtained from the database that exhibit a high value of the bond-order parameter q_6 (see Figure 3 of the main text). The $N = 38$ cluster corresponds to a truncated octahedron.

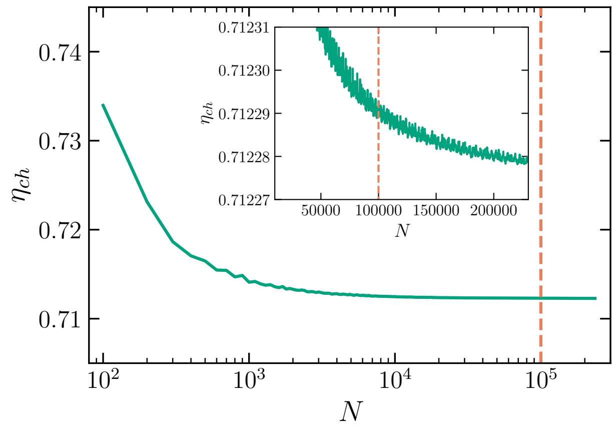

FIG. S6. Packing fraction η_{ch} as a function of the number of points N_{points} used to calculate the convex hull. The inset shows that for $N_{points} = 10^5$ the precision in the packing fraction is on the fourth decimal digit.

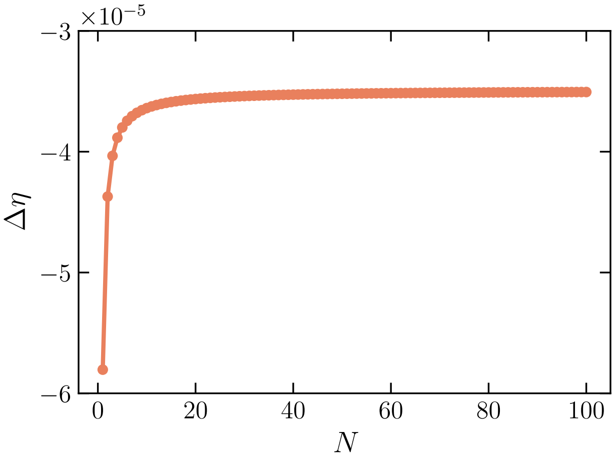

FIG. S7. Difference in packing fraction $\Delta\eta$ between the analytical value calculated on the linear spherocylinder and the estimated value calculated by means of the convex hull as a function of the number of particles N.

Clusters from truncated polyhedra

In Table S3, we report the main characteristics of clusters that exhibit a higher convex-hull packing fraction η_{ch} than the linear configuration at a given N, as shown in Figure 3 of the main text. The following characteristics are listed: N, which refers to the total number of colloids; *ID*, name of the cluster for truncated tetrahedra and bipyramids as defined in the main text; η_{ch}, the packing fraction of the convex hull that encloses the cluster of spheres; *Faces*, the number of faces in the cluster, which is defined as the minimum number of planes that can be constructed on the surface of the cluster; *Vertices*, number of vertices of the cluster, where each vertex is defined as the sphere at the intersection of three or more planes; N_{inner}, the total number of particles that do not belong

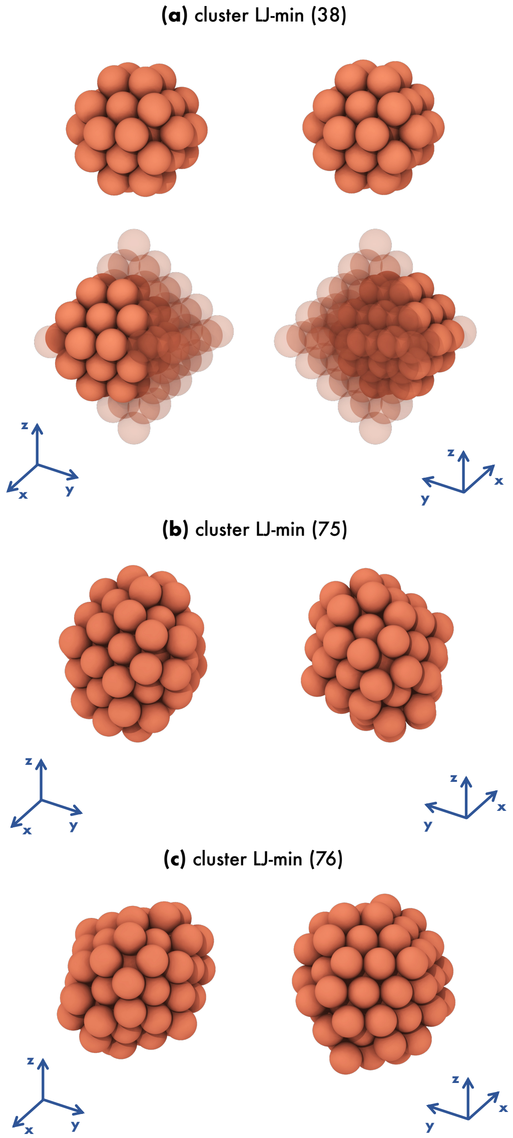

FIG. S8. Typical clusters from the database that exhibit a high value of the bond order parameter q_6 from different perspectives. The $N = 38$ cluster corresponds to a truncated octahedron, transparent spheres are the ones that have been removed from the original regular octahedron.

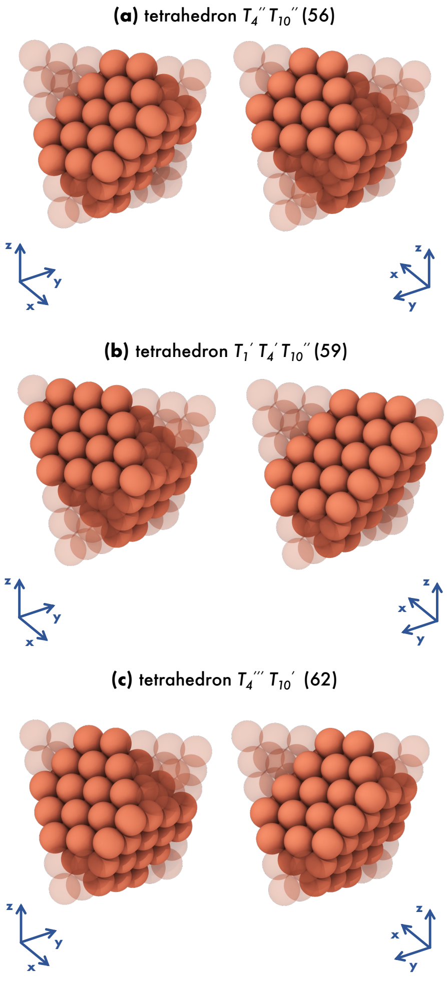

FIG. S9. Configurations of truncated tetrahedra that exhibit a higher convex-hull packing fraction η_{ch} than the linear arrangement at the corresponding N from various perspectives, see also Figure 3 of the main text. The transparent spheres indicate those that have been eliminated from the original regular polyhedron.

to any of the planes on the surface of the cluster; N_{face}, the total number of particles that belong to the planes on the surface of the cluster; N_{edge}, the total number of particles located at the intersection of two planes; η_{lin}, the packing fraction of the linear arrangement of spheres at the same N; *Snapshots*, which provides a reference to the figure reporting the respective cluster; and *3D View* which gives the short name of HTML files from the Supplemental Material that contain three-dimensional visualization of the cluster.

Comparison of the packing fraction η_{ch} between different stackings

Along with the face-centered cubic (FCC) arrangement, it was proven that for an infinite number of spheres there exists an infinite number of other stackings that achieve the same packing efficiency of $\approx 74\%$ [6]. The latter are better known as Barlow stackings [7], and they are all based on variations of the three ways of accommodating an hexagonal layer on top of another. Each layer can be identified by the letters 'A', 'B' and 'C', with different combinations encoding for different stackings. The most regular and well known stacking are the hexagonal-closed packing (HCP) and the FCC arrangements with repeated sequences 'AB' and 'ABC' respectively. Here, we extend the study of the FCC-based structures presented in the

Truncated Tetrahedron										
N	ID	η_{ch}	Faces	Vertices	N_{inner}	N_{face}	N_{edge}	η_{lin}	Snapshots	3D View
56	$T_4''T_{10}''$	0.67102	8	11	8	18	19	0.67066	S9(a)	TT56
59	$T_1'T_4'T_{10}''$	0.67047	8	11	8	21	19	0.67045	S9(b)	TT59
62	$T_4'''T_{10}'$	0.67134	8	12	9	23	18	0.67027	S9(c)	TT62
Truncated Octahedron										
58		0.67120	10	14	10	16	18	0.67052	S10(a)	TO58
59		0.67179	9	11	10	16	22	0.67045	S10(b)	TO59
60		0.67237	8	8	10	16	26	0.67039	S10(c)	TO60
64		0.67030	12	19	13	15	19	0.67016	S10(d)	TO64
65		0.67111	12	20	13	19	13	0.67010	S10(e)	TO65
66		0.67164	11	17	13	19	17	0.67005	S10(f)	TO66
67		0.67241	11	18	13	21	15	0.67000	S10(g)	TO67
68		0.67266	9	11	13	19	25	0.66995	S10(h)	TO68
69		0.67339	9	12	13	21	23	0.66990	S10(i)	TO69
70		0.67387	8	9	13	21	27	0.66986	S10(j)	TO70
Truncated Bipyramid										
61	$B_{10}'B_{20}'$	0.67146	8	9	9	13	30	0.67033	S11(a)	TB61
67	$B_4'B_{20}'$	0.67171	8	9	10	18	30	0.67000	S11(b)	TB67

TABLE S3. List of the main characteristics of clusters that exhibit a higher convex-hull packing fraction η_{ch} than the linear configuration at a given N.

main text by building clusters made of 6 layers with the '$ABABAB$' sequence corresponding to HCP, '$ABABAC$' (seq 1) and '$ABACBC$' (seq 2) [8]. In Fig. S12(a) we show the convex hull packing fraction η_{ch} of HCP-based pyramid (P_{HCP}) and bipyramid (B_{HCP}) clusters compared to the corresponding structures based on an FCC arrangement. We find that the different stacking introduced by the HCP arrangement is not as favorable as the one formed by the FCC for packing finite structures. A similar situation occurs with the other stackings, as shown in Fig. S12(b), where we show the η_{ch} of the pyramids based on the seq 1 and seq 2 (dashed symbols). Moreover, we perform irregular cuts to clusters based on HCP, seq 1 and seq 2, finding that all of them have a significantly lower η_{ch} compared to η_{lin}. We report in Table S4 the corresponding clusters with their convex-hull packing fraction η_{ch} when compared with the linear convex-hull packing fraction η_{lin} and the *3D View* which gives the short name of HTML files (Supplementary Data 1) that contain the interactive three-dimensional visualization of the cluster.

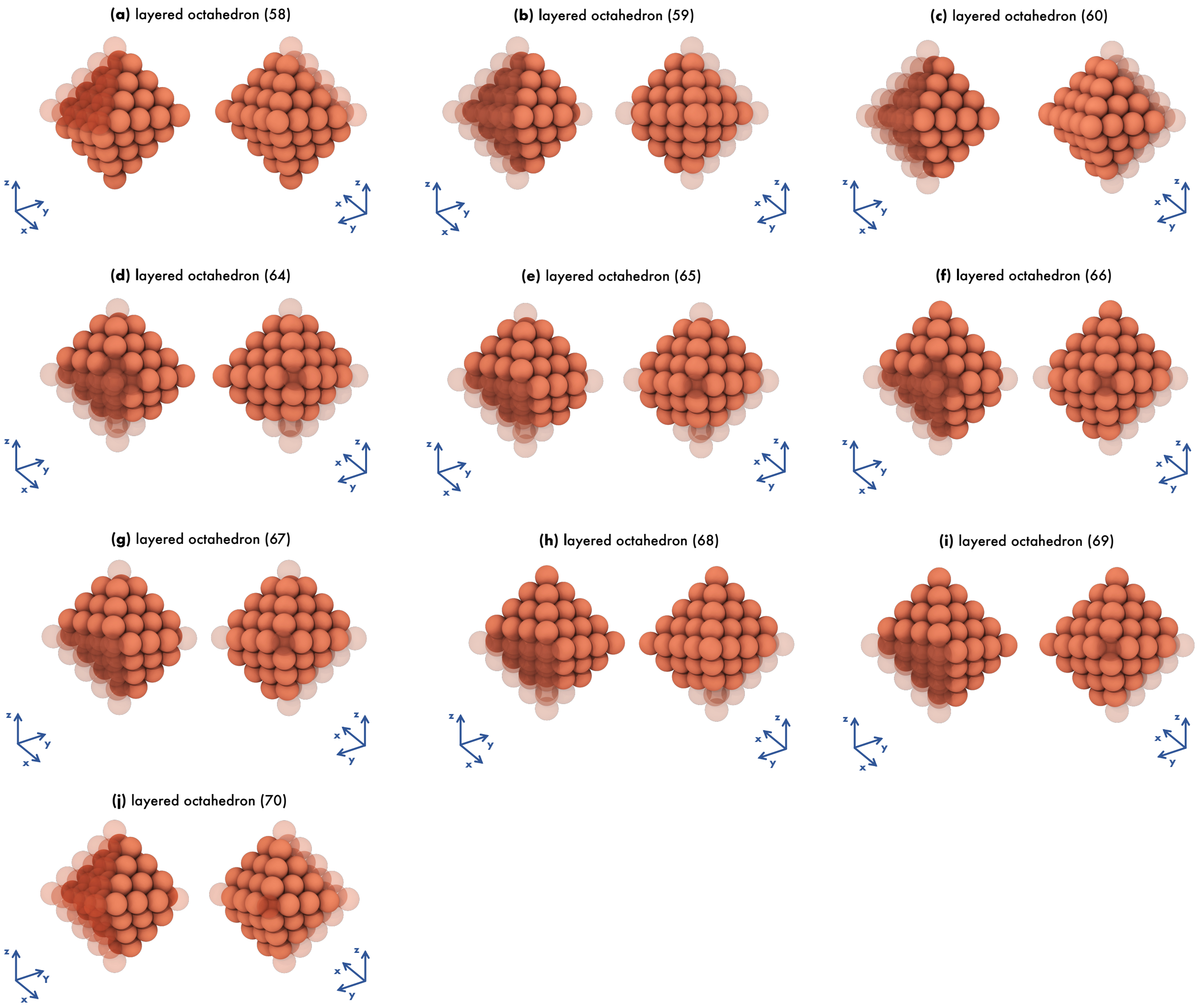

FIG. S10. Configurations of truncated octahedra that exhibit a higher convex-hull packing fraction η_{ch} than the linear arrangement at the corresponding N from various perspectives, see also Figure 3 of the main text. The transparent spheres indicate those that have been eliminated from the original regular polyhedron.

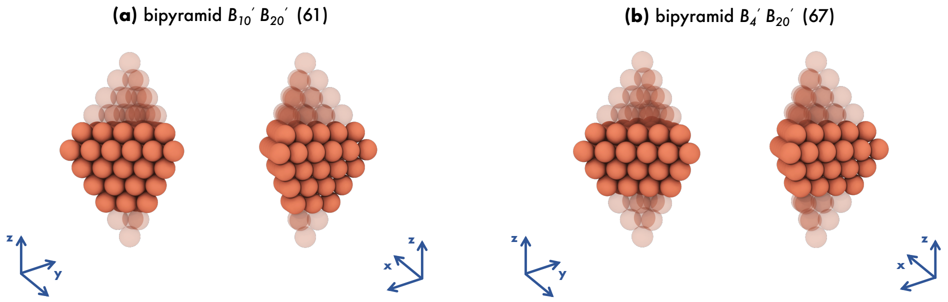

FIG. S11. Configurations of truncated bipyramids that exhibit a higher convex-hull packing fraction η_{ch} than the linear arrangement at the corresponding N from various perspectives, see also Figure 3 of the main text. The transparent spheres indicate those that have been eliminated from the original regular polyhedron.

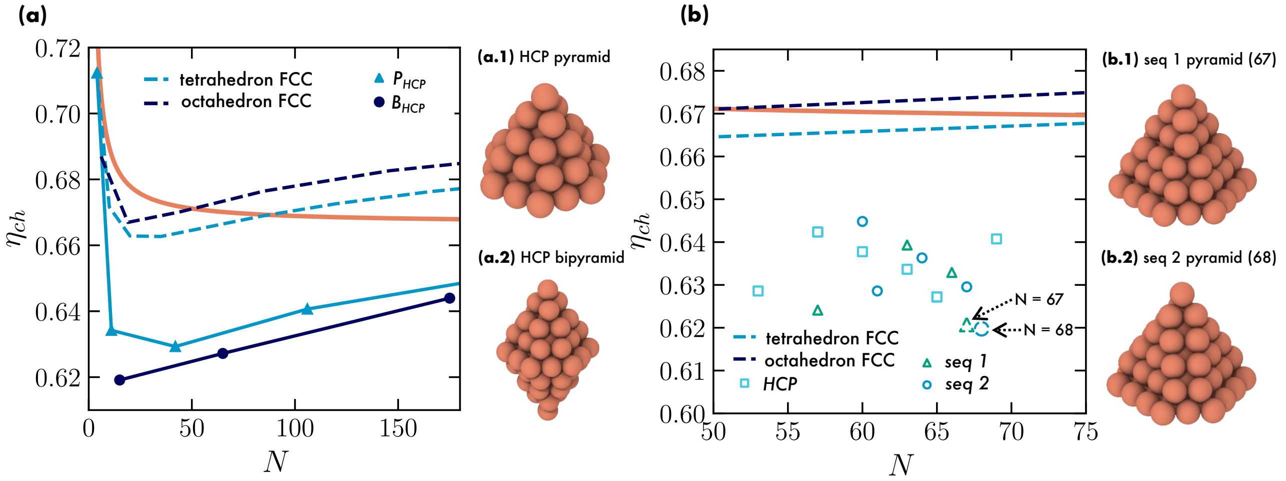

FIG. S12. Packing fraction η_{ch} of clusters of spheres in their convex hull as a function of N for (a) HCP-based (ABABAB stacking) pyramids and bipyramids, and for (b) irregularly cut HCP-based, ABABAC- and ABACBC-based polyhedra as compared to tetrahedra and octahedra based on a FCC staking of the particle layers (dashed lines). The ideal linear packing fraction $\eta_{lin}(N)$ is also reported (orange line).

HCP			
N	η_{ch}	η_{lin}	3D View
65	0.62719	0.67010	BHCP65
106	0.64065	0.6687	PHCP106
69	0.64074	0.66990	TPHCP69
72	0.63452	0.66977	TPHCP72
77	0.65889	0.66956	TPHCP77
53	0.62859	0.67089	TBHCP53
57	0.64236	0.67059	TBHCP57
60	0.63776	0.67039	TBHCP60
63	0.63366	0.67021	TBHCP63
Sequence 1 *ABABAC*			
57	0.62412	0.67059	Seq1_57
63	0.63932	0.67021	Seq1_63
66	0.63291	0.67005	Seq1_66
67	0.62074	0.67000	Seq1_67
Sequence 2 *ABACBC*			
60	0.64482	0.67039	Seq2_67
61	0.62859	0.67033	Seq2_61
64	0.63631	0.67016	Seq2_64
67	0.62955	0.67000	Seq2_67
68	0.64482	0.66995	Seq2_68

TABLE S4. List of clusters and corresponding convex-hull packing fraction η_{ch} reported in Fig. S12b with stacking based on HCP (ABABAB), sequence 1 (ABABAC) and sequence 2 (ABACBAC) and corresponding ideal linear packing fraction η_{lin} for a certain number of colloids N.

* s.marinaguilar@uu.nl
† f.camerin@uu.nl
‡ h.r.vutukuri@utwente.nl
§ m.dijkstra@uu.nl

[1] A. Stukowski, Modelling and simulation in materials science and engineering **18**, 015012 (2009).
[2] A. Stukowski, Jom **66**, 399 (2014).
[3] E. Bitzek, P. Koskinen, F. Gähler, M. Moseler, and P. Gumbsch, Physical review letters **97**, 170201 (2006).
[4] M. Mravlak, T. Kister, T. Kraus, and T. Schilling, The Journal of Chemical Physics **145**, 024302 (2016).
[5] C. B. Barber, D. P. Dobkin, and H. Huhdanpaa, ACM Transactions on Mathematical Software (TOMS) **22**, 469 (1996).
[6] T. Hales, M. Adams, G. Bauer, T. D. Dang, J. Harrison, H. Le Truong, C. Kaliszyk, V. Magron, S. McLaughlin, T. T. Nguyen, *et al.*, in *Forum of mathematics, Pi*, Vol. 5 (Cambridge University Press, 2017) p. e2.
[7] W. Barlow, Nature **29** (1884).
[8] R. M. Thompson and R. T. Downs, Acta Crystallographica Section B: Structural Science **57**, 766 (2001).
