# Supplementary material for: A colloidal viewpoint on the sausage catastrophe and the finite sphere packing problem
Source: Nat Commun. 2023 Nov 30;14:7896. doi: 10.1038/s41467-023-43722-0 (PMC10689752; doi:10.1038/s41467-023-43722-0)
Supplement: Supplementary file 3 — Description of Additional Supplementary Files [file 41467_2023_43722_MOESM3_ESM.pdf]

Description of Additional Supplementary Files

File name: html.zip (Supplementary Data 1)
Description:
Interactive HTML files containing the three-dimensional visualization of the clusters reported in the Supplementary Information file.

File name: movieS1.mp4 (Movie S1)
Description:
Upper panel: Time-lapsed overlaid fluorescence and bright-field microscopy movie illustrating the linear arrangement or sausage for 9 particles in a low-tension vesicle. Note that the first 5 seconds of the movie only show the membrane. The movie was recorded at 5 fps and played back at 10 fps.
Bottom panel: Corresponding movie for a simulation of the linear arrangement of 9 particles.

File name: movieS2.mp4 (Movie S2)
Description:
Upper panel: Time-lapsed overlaid 2D confocal and bright-field microscopy movie demonstrating the coexistence between the sausage and plate arrangements for 4 particles. The movie was recorded at 2 fps and displayed at 50 fps. Bottom panel: Corresponding simulation movie for a bistable state point between linear and plate configuration for 4 particles.

File name: movieS3.mp4 (Movie S3)
Description:
Upper panel: Time-lapsed 2D overlaid fluorescence and bright-field microscopy movie showing the transition from linear to cluster arrangement by inducing osmotic imbalances in a vesicle with 5 particles. Bottom panel: Corresponding simulation movie for a transition from linear to cluster arrangement by changing the external solvent density.
