# Supplementary material for: A colloidal viewpoint on the sausage catastrophe and the finite sphere packing problem
Source: Nat Commun. 2023 Nov 30;14:7896. doi: 10.1038/s41467-023-43722-0 (PMC10689752; doi:10.1038/s41467-023-43722-0)
Supplement: Supplementary file 4 — Supplementary Data 1 [file 41467_2023_43722_MOESM4_ESM.zip › html/TT56_Truncated_Tetrahedron_56_particles.html]

Truncated Tetrahedron 56 particles


## Truncated Tetrahedron 56 particles

4 vertices truncated: 2 removed layers in two vertices and 3 layers in other two vertices

Made using  Visual colloids.
