# Supplementary material for: A colloidal viewpoint on the sausage catastrophe and the finite sphere packing problem
Source: Nat Commun. 2023 Nov 30;14:7896. doi: 10.1038/s41467-023-43722-0 (PMC10689752; doi:10.1038/s41467-023-43722-0)
Supplement: Supplementary file 4 — Supplementary Data 1 [file 41467_2023_43722_MOESM4_ESM.zip › html/TB67_Truncated_Bipyramid_67_particles.html]

Truncated Bipyramid 67 particles


## Truncated Bipyramid 67 particles

2 and 4 layers removed from each of the acute vertices

Made using  Visual colloids.
